# Surgery

**Published:** 1899-06

**Authors:** C. A. Morton


### SURGERY.

The best method of dealing with the **pedicle** after the **removal of uterine fibroids** by cœliotomy is still undecided.

Mr. Bland Sutton, in a paper read before the Obstetrical Society of London,[3] on 28 cases of hysterectomy with intraperitoneal treatment of the pedicle, strongly advocates that method. His results—only 2 deaths in 28 cases—are very good; but we must remember that he lays it down most distinctly that all fibroids should be removed just as all ovarian cysts ought to be, and therefore he is likely to have a series of less formidable tumours than those who only operate when the tumour is growing very large, or causes considerable distress or serious hemorrhage. His contention that his mortality in these cases is as low as after ovariotomy is hardly correct, for—as pointed out by one of the speakers in the debate on

[3] *Tr. Obst. Soc. Lond.*, 1898, xxxix. 292.

his paper—7 per cent. for ovariotomy is rather high at the present time, and if operating in all cases gives a mortality of 7 per cent. it would probably be considerably higher with surgeons who only operate on very large tumours, or those causing pressure symptoms and hemorrhage, and it is only on such tumours that the majority of surgeons and gynæcologists operate.

Moreover, Mr. Meredith (one of the surgeons to the Samaritan Hospital), at the same meeting of the society, referred to [1] his own results with the extra-peritoneal method, and they give a lower mortality than Mr. Bland Sutton's. Mr. Meredith had a run of 30 cases treated by the extra-peritoneal method without a death, and two series of 40 and 47 cases respectively with but two deaths in each, and 83 recoveries out of a total of 90 cases—results which, he says, "have not as yet, so far as I know, been equalled by advocates of the intra-peritoneal method;" and Mr. Meredith is not a surgeon who operates on uterine fibroids without special reason, and therefore would not have such simple cases to deal with as Mr. Bland Sutton. Mr. Knowsley Thornton (also surgeon to the Samaritan Hospital), in an article on hysterectomy,[2] says that, putting on one side cases in which large fibroid tumours burrow in the broad ligament or under the parietal peritoneum as a specially difficult and dangerous class of cases, in his practice cases treated by the serre-nœud had a mortality of only 8 per cent., as against 50 per cent. in cases treated by the intra-peritoneal method.

There is always a risk of injury to the ureter in the intra-peritoneal method; and, if the uterine arteries are not efficiently secured, of hemorrhage also, and the ligation of the uterine arteries may not be an easy matter in cases in which the tumour burrows in the broad ligament, and in such cases the ureters would be in special danger. Although in the extra-peritoneal method a slough is produced by the wire of the serre-nœud, yet this slough is not offensive, but tends to dry up if properly treated, nor is there any risk of peritonitis from it, for if it is rightly placed with regard to the peritoneum the peritoneal cavity is safely shut off before suppuration begins around the constricted portion of the pedicle. There certainly is no evidence that peritonitis has ever been produced by dragging on the pedicle. Hemorrhage after the operation, from slipping of the wire, would be an accident due to its improper application. In the intra-peritoneal method there is always a possibility of infection of the peritoneum from the opened uterine cavity, and some surgeons have applied the cautery or strong antiseptic to the cervical canal to prevent this. For in writing of the intra-peritoneal method, I refer to that originally practised by Baer,[3] and now employed by Bland Sutton, in which

[1] *Tr. Obst. Soc. Lond.*, 1898, xxxix. 306.
[2] Clifford Allbutt and Playfair's *System of Gynæcology*, 1896.
[3] *N. York J. Gynæc. & Obst*, 1892, ii. 986.

the cervix is left without ligation, and the bleeding from its surface controlled by previous ligation of the uterine arteries. Complete removal of the uterus, with its greater risk of injury to the ureters, seems unnecessary. But the extra-peritoneal method necessitates a more tedious recovery than the intra-peritoneal method, and this is really the chief objection to it. On the other hand, to rapidly complete an operation for the removal of a very large tumour, or to avoid the risk of injury to the ureters in cases in which the tumour has opened up the broad ligaments near the cervix and thus left little room for ligation of the uterine arteries, the extra-peritoneal method has distinct advantages.

The publication of one case treated by either method is, of course, of little value as an argument for its general adoption; but to show that the results of the extra-peritoneal method are not so dreadful as some writers have represented, I will briefly refer to some details of one of my own cases.

The tumour reached across the abdomen from one anterior spine to the other, and extended two inches above the umbilicus. After removal it weighed five pounds. It was complicated with a small ovarian cyst. The body of the uterus was buried in the tumour, and I applied the serre-nœud two inches above the vaginal attachment. The operation was performed on July 18th, in the Hospital for Children and Women. She never had a bad symptom. By the 21st the constricted portion of the pedicle was getting quite hard and dry, not "hanging out from the abdominal wall with foul pus discharging from the stump," as the usual condition of the pedicle has been recently described.[1] The serre-nœud handle was removed on the 21st, and the wire and pins later. On September 4th she was discharged; the sinus had not healed, but it closed some time after.[2]

The more recently published results of **operations for the removal of malignant tumour** are of much value, especially with regard to the breast and the tongue, and they may be said to be distinctly encouraging; indeed we are now able to speak of long periods of non-recurrence after the removal of malignant tumour in certain parts, in a way never thought of some years ago. This is due to early and free removal.

The average duration of life in **cancer of the breast** not operated on is 2½ years from the time when it is first noticed. The risk of operation is very small. Watson Cheyne[3] has operated on 61 cases with a mortality of only 1.6 per cent., and Halsted on 50 with no death. All these operations were

[1] *Bristol M.-Chir. J.*, 1899, xvii. 9.

[2] Another case, also treated by the serre-nœud, is referred to in my remarks at the May meeting of the Bristol Medico-Chirurgical Society.

[3] *Brit. M. J.*, 1896, i. 390.

extensive ones, including removal of glands. They were, of course, all aseptic. With a very thorough removal of the disease together with the glands, whether palpable before operation or not, out of the 61 cases operated on by Watson Cheyne up to three years ago no less than half are cured.[1] Although the term "cure" as applied to these cases only means absence of recurrence for three years or longer, the further history of the eleven "cured" cases which Watson Cheyne reported in 1896 shows that in only one has there so far been any recurrence of the disease, and in that case it was probably a fresh development, as the patient is reported to have died of cancer of the intestine six years after the breast operation. Halsted (who is Professor of Surgery in the Johns Hopkins Hospital, Baltimore) has recently given us[2] the results of his operations for cancer of the breast up to April, 1898. He says that "of the 76 cases operated upon three or more years ago, 31 (41 per cent.) are living without local recurrence or signs of metastases." His method of operating is even more extensive than Watson Cheyne's, for he removes the greater part of the pectoralis major muscle in every case. Mr. Butlin has practised the method for the last few years, and has had a remarkably successful series of cases, for he had eight cures out of thirteen cases operated on.[3] He regards this as a very fortunate series, and says that he does not expect "that the next or many future series of thirteen cases will yield nearly such good results"; but it shows what operation can do for cancer of the breast, especially when we note that Butlin says that the cases were not obtained early for operation; "there is scarcely one in which the disease was not known to have existed several months, and there are many in which it had existed for one, two, three, or more years. There were many in which the axilla was full of cancerous glands, and several in which the primary tumour was ulcerated." Warren[4] has also had 17 cures in 72 operations, or about 25 per cent. I have not yet followed up the histories of all the patients I have operated on for cancer of the breast—and I have always operated in the thorough way that Watson Cheyne does,—but I know of a few cases free from disease more than three years from the operation. In one case, after three years from the operation, there has twice been very slight local recurrence in the neighbourhood of the scar. These recurrent growths have been removed.

The chance, then, of prolongation of life, and even cure of the disease, is so good, that we can unhesitatingly advise operation, which must be of the thorough or so-called "complete" kind which Watson Cheyne practises, and which he has described in his Lettsomian Lectures,[5] and has more recently referred to his paper in the *Lancet*.[6]

[1] *Lancet*, 1899, i. 756. [2] *Tr. Am. Surg. Ass.*, 1898, xvi. 162.

[3] *St. Barth. Hosp. Rep.*, 1899, xxxiv. 49.

[4] *Boston M. & S. J.*, 1898, cxxxix. 186. [5] *Loc. cit.* [6] *Loc. cit.*

The only question which remains to be considered is, What are the **contra-indications to operation?** Watson Cheyne gives them as follows:[1] 1. Cases of cancer *en cuirasse.* 2. Cases where there is a large mass in the axilla involving the nerves. 3. Cases where large glands can be felt above the clavicle. 4. All cases where secondary cancers already exist elsewhere. He has removed a portion of the axillary vein several times when involved in gland growth, and once a portion of the artery and vein, without any harm resulting. I have myself also more than once removed portions of the axillary vein with the gland-growth without any harm resulting. Halsted[2] does not now consider enlargement of the supra-clavicular glands a contra-indication to operation, and he clears out the supra-clavicular region thoroughly in every case. He has records of two cases on which he operated for removal of supra-clavicular glands which appeared after the breast and axillary contents had been removed, in which there was no recurrence three and a half years after the supra-clavicular glands were excised. Halsted says: "In one of these latter cases I considered the prognosis desperately bad, at both the infra- and supra-clavicular operations. At the first operation the cancer had infiltrated the axillary fat diffusely, and could with difficulty be separated from the subclavian vein; at the second operation the same desperate state of affairs was encountered in the neck."

The results of operations for **cancer of the tongue** have been considered in recent papers by Watson Cheyne[3] and Butlin.[4] Watson Cheyne, from a study of the statistics of some Continental surgeons, comes to the conclusion that the mortality of excision of cancer of the tongue may be estimated at 15 to 20 per cent., but some surgeons have had a much lower death-rate in a certain series of cases. Thus Kocher had only one death in his last series of 28 cases, and Volkmann only two deaths in 91 cases. Kocher's cures amounted to 18 per cent., Koenig's to 12 per cent. Mr. Butlin's results are particularly encouraging. He has operated on 102 cases, with a mortality of 10 per cent., and 20 per cent. of cures. His private operations—or in other words his operations on cases at an earlier stage than the hospital ones—give the splendid result of only one death in 49 cases, and 13 cures. These operations are included in the 102 already given, and this result is an illustration of the desirability of operating for cancer at the earliest possible moment.

In cancer of the tongue we have a disease which proves fatal without operation in a year to eighteen months, and towards the end is a terribly painful and distressing disease. At a moderate risk—5 per cent. mortality in early cases limited to

[1] *Brit. M. J.*, 1896, i. 387. [2] *Loc. cit.*
[3] *Brit. M. J.*, 1896, i. 449. [4] *Ibid.*, 1898, i. 541.

the tongue, but with 15 to 20 per cent. mortality in extensive cases, in which the disease has extended to the floor of the mouth, palate and tonsils—the disease can be removed with a chance of cure in 20 per cent. of cases (and even more in earlier cases), a chance of prolongation of life by many months in almost 95 per cent. of early and 80 to 85 per cent. of extensive cases.

If recurrence takes place after operation, in many cases it is not in the mouth, where the disease is so distressing, but in the glands of the neck, and such recurrence in most cases admits of removal.

The lesson which a study of recent literature on the treatment of cancer of the tongue teaches most emphatically is, that at the very earliest moment the growth should be removed, and that weeks must not be lost in treating an ulcer of doubtful nature by iodide, when the diagnosis can be established in a few days by the microscopic examination of a portion of tissue snipped off the edge of the ulcer under cocaine.

C. A. MORTON.

